# Activity of mevalonate pathway inhibitors against breast and ovarian cancers in the ATP-based tumour chemosensitivity assay

**DOI:** 10.1186/1471-2407-9-38

**Published:** 2009-01-28

**Authors:** Louise A Knight, Christian M Kurbacher, Sharon Glaysher, Augusta Fernando, Ralf Reichelt, Susanne Dexel, Uwe Reinhold, Ian A Cree

**Affiliations:** 1 Translational Oncology Research Centre, Queen Alexandra Hospital, The Pathology Centre, Portsmouth, PO6 3LY, UK; 2 L.a.n.c.e. Inc, Friedensplatz 16, 53111 Bonn, Germany; 3 Department of Dermatology, Medical Center Bonn-Friedenplatz, Friedensplatz 16, 53111 Bonn, Germany

## Abstract

Previous data suggest that lipophilic statins such as fluvastatin and N-bisphosphonates such as zoledronic acid, both inhibitors of the mevalonate metabolic pathway, have anti-cancer effects *in vitro *and in patients. We have examined the effect of fluvastatin alone and in combination with zoledronic acid in the ATP-based tumour chemosensitivity assay (ATP-TCA) for effects on breast and ovarian cancer tumour-derived cells. Both zoledronic acid and fluvastatin showed activity in the ATP-TCA against breast and ovarian cancer, though fluvastatin alone was less active, particularly against breast cancer. The combination of zoledronic acid and fluvastatin was more active than either single agent in the ATP-TCA with some synergy against breast and ovarian cancer tumour-derived cells. Sequential drug experiments showed that pre-treatment of ovarian tumour cells with fluvastatin resulted in decreased sensitivity to zoledronic acid. Addition of mevalonate pathway components with zoledronic acid with or without fluvastatin showed little effect, while mevalonate did reduced inhibition due to fluvastatin. These data suggest that the combination of zoledronic acid and fluvastatin may have activity against breast and ovarian cancer based on direct anti-cancer cell effects. A clinical trial to test this is in preparation.

## Background

**Figure 1:**
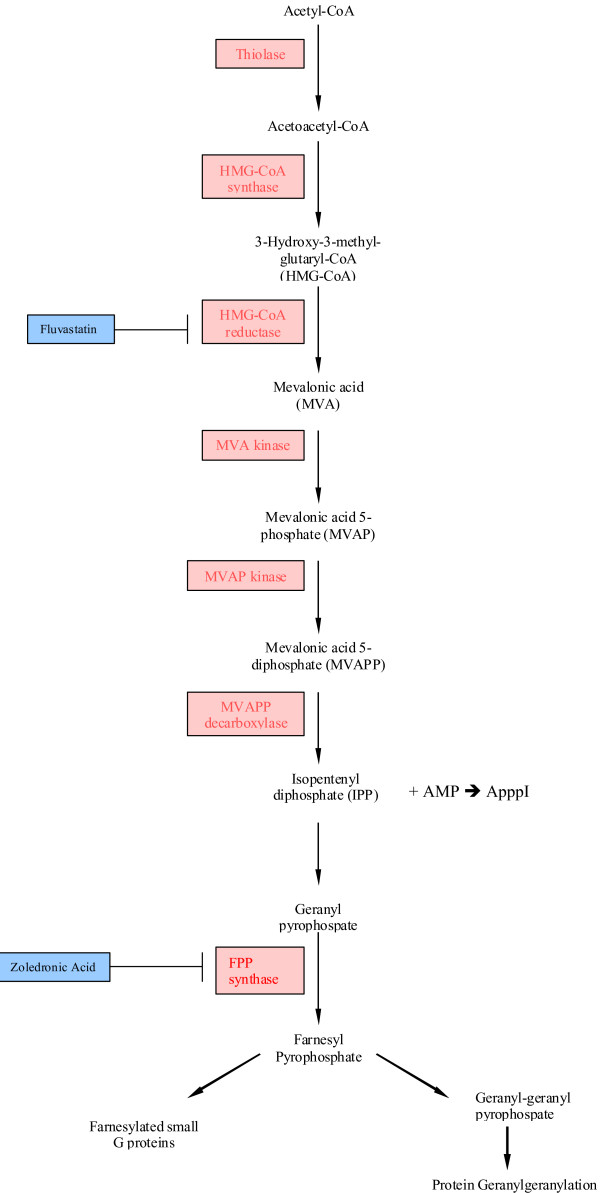
**Diagram of the mevalonate pathway**. N-bisophosphonates inhibit FPP-synthase, leading to accumulation of IPP, which generates ApppI from AMP. ApppI has been found to be toxic to cells [19], while statins inhibit HMG-CoA reductase.

The mevalonate pathway performs several key functions within cells leading to the production of sterols such as cholesterol essential to membrane formation, and to the post-translational modification by prenylation of proteins such as Ras and other small G proteins, which are important second messengers of growth signals from membrane growth factor receptors [1]. The process of prenylation involves farnesylation and geranylgeranylation from the mevalonate metabolite farnesyl pyrophosphate (FPP) as shown in figure 1. While farnesylation is usually required for translocation of Ras to the cell membrane during its activation [2], N-Ras and K-Ras can be geranylgeranylated in the presence of farnesyl transferase inhibitors (FTIs), providing a rationale for the limited clinical activity of these agents [3,4]. Ras signalling is essential to many cancers, either as part of activated growth receptor pathways or by the acquisition of activating mutations during carcinogenesis. There is therefore considerable interest in inhibiting the mevalonate pathway to treat cancers.

The mevalonate pathway can be interrupted by existing drugs at several levels. As mevalonate is synthesized from 3-hydroxy-3-methylglutaryl coenzyme A (HMG-CoA), HMG-CoA inhibitors such as the statins reduce the entry of mevalonate into the pathway. This may explain the observed effects of statins, normally used to lower cholesterol levels, on the possible survival benefit in patients with non-small cell lung cancer (NSCLC) following chemotherapy [5], and other effects in a wide variety of tumour types. The newer N-bisphosphonates such as ibandronate (Roche) and zoledronic acid (Novartis) are inhibitors of farnesyl pyrophosphate (FPP) synthase, and therefore reduce the amount of both FPP and GGPP available for prenylation of Ras [6,7]. Growth inhibitory effects of these agents have been noted in cancer cell lines and in tumour-derived cells [7,8]. Finally, FTIs prevent the farnesylation of Ras and have effects *in vitro *on cell growth, though their effect in cancer patients has been disappointing [9], and we have seen little effect in tumour derived cells. This may reflect the redundancy between farnesylation and geranylgeranylation, such that inhibition of one is insufficient to prevent the action of the other [3,4].

We have previously shown direct activity of the N-bisphosphonate zoledronic acid in an ATP-based tumour chemosensitivity assay (ATP-TCA) against a variety of tumour types, including breast and ovarian cancer [8]. We have now extended these studies to examine the effect of fluvastatin alone and in combination with zoledronic acid against ovarian and breast cancer *in vitro*.

## Methods

### Tumour samples

A total of 31 tumours were tested in this study, comprising of 9 primary breast and 22 pre-treated (mostly with platinum based chemotherapy) ovarian carcinomas. The median age was 50 (range 41–78) and 58 (range 33–86) respectively. Samples were obtained from laboratories located in Germany and the UK. In each case only tissue surplus to diagnostic requirements was used for research, in accordance with local research ethics committee approval. All patients gave informed consent for the research use of their tissue.

### Drugs

Zoledronic acid (hydrated sodium salt) was obtained from Novartis (Basel, Switzerland), and fluvastatin (344095-25) was obtained from VWR International (Leicestershire, UK). Both drugs were diluted in complete assay media (CAM) to concentrations thought to be achievable clinically. Zoledronic acid was tested at 2.2 – 69.0 μM (100% test drug concentration, TDC = 34.5 μM). Fluvastatin was tested at two concentration ranges: A = 0.1 – 2.7 μM (100% TDC = 1.4 μM) and B = 0.7 – 23.0 μM (100% TDC = 11.5 μM) in the German and Portsmouth laboratories respectively. Combinations of zoledronic acid and fluvastatin were tested by simultaneous addition.

### ATP-TCA

The ATP-TCA was performed as previously described [8,10,11]. Briefly, tumour cells were dissociated from solid tumour by collagenase (Sigma; C8051) digestion and extracted either from the resulting cell suspension, or directly from ascites, by density centrifugation over Ficoll (Sigma; 1077-1). Cells were re-suspended in serum-free CAM (DCS Innovative Diagnostik Systeme, Hamburg, Germany) and plated in 96-well polypropylene plates (Corning Life Sciences, High Wycombe, UK) at 20,000 solid tumour-derived or 10,000 ascites-derived cells per well in 100 μl CAM.

Drugs were added in a further 100 μl to triplicate wells at serial dilutions corresponding to 6.25–200% of a clinically achievable test drug concentration (TDC) estimated from pharmacokinetic data, including the degree of protein binding [10]. Each plate contained two rows of control wells: one contained medium-only (MO), while the other contained a maximum inhibitor (MI) control which kills all cells present. The plates were incubated for 6 days at 37°C, with 5% CO_2 _and 99% humidity. At the end of the incubation period, cells were lysed by addition of cell extraction reagent (DCS Innovative Diagnostik Systeme). A 50 μl aliquot of the lysate from each well was added to the corresponding wells of a white 96-well microplate (Thermo Life Sciences, Basingstoke, UK), followed by addition of 50 μl luciferin-luciferase reagent. Light output is directly proportional to the amount of ATP present and was measured in a luminometer (MPLX; Berthold Detection Systems, Hamburg, Germany). The percentage inhibition at each of the six drug concentrations tested was calculated in relation to controls as: (1 - (test-MI)/(MO-MI))*100. The IC50 and IC90 were calculated by the trapezoidal rule, and a natural logarithmic sum index (Index_SUM_) was obtained by direct addition of the percentage survival at each concentration tested (Index_SUM _= 600-Σ (%Inhibition_6.25...200_) as this has been shown to provide a better indication of sensitivity or resistance to different drugs in different tumour types than other ATP-TCA parameters [12]. Total inhibition results in an Index_SUM _of 0 (sensitivity), while no inhibition at any concentration produces an index of 600 (resistance).

### Data analysis

The ATP-TCA results were entered into an ACCESS (Microsoft) database for further analysis. Statistical tests were performed using non-parametric methods. Additive or synergistic effects were assessed using the methods described by Poch *et al*., [13]. When synergy was indicated by the Poch method, Chou & Talalay [14] analysis was used to evaluate combinational effects. The combinational indicies (CI) at 50% and 90% were calculated in the following way:

CI_A+B _= [(D_A/A+B_)/D_A_]+ [(D_B/A+B_)/D_B_]+ [α(D_A/A+B_)/D_A_D_B_]

Where CI_A+B _= CI for fixed effect (F = 50% or 90%) for the combination of cytotoxic A and cytotoxic B; D_A/A+B _= concentration of cytotoxic A in the combination A+B giving an effect F; D_B/A+B _= concentration of cytotoxic B in the combination A+B giving an effect F; D_A _= concentration of cytotoxic A alone giving an effect F; D_B _= concentration of cytotoxic B alone giving an effect F. α = parameter with value 0 when A and B are mutually exclusive and 1 when A and B are mutually non-exclusive. The CI index indicated synergism <0.8; additivity >0.8 and <1.2 and antagonism >1.2 [15].

### Sequential studies

Ovarian tumour derived cell suspensions were added to round-bottomed polypropylene 96-well plates prepared with 100 μl CAM pre-conditioned with either fluvastatin (2.9 μM) or zoledronic acid (8.6 μM). The corresponding drug plates were prepared with CAM and single agent fluvastatin or zoledronic acid at six dilutions (6.25–200%) of the test drug concentration in triplicate. Each plate included two internal controls (MI and MO). Both the cell plates and the drug plates were incubated at 37°C in 5% CO_2 _for 24 hours. Following pre-incubation, cells were pelleted via centrifugation and the media discarded. The contents of the fluvastatin drug plate were transferred to the zoledronic acid pre-conditioned cells and the contents of the zoledronic acid drug plate were transferred to the fluvastatin pre-conditioned cells. The cell suspensions were gently mixed. The plates containing both cells and drugs at six dilutions were incubated for a further 5 days at 37°C in 5% CO_2_. After the 5 day incubation the cells were lysed and the ATP concentration measured as described previously. The results were assessed as previously described and compared to the ATP-TCA single agent results for each drug.

### Substrate replacement studies

Substrate replacement experiments were conducted using Farnesyl diphosphate (FPP; Echelon Biosciences Inc, Salt Lake City, UT, USA), Mevalonate (Sigma Chemical Co Ltd, Poole, UK), Farnesol (Sigma), and Geranylgeraniol (Sigma) by simultaneous addition, or pre-incubation using a static concentration or double dilution as indicated in the results section.

## Results

### Effect of single agents

**Figure 2:**
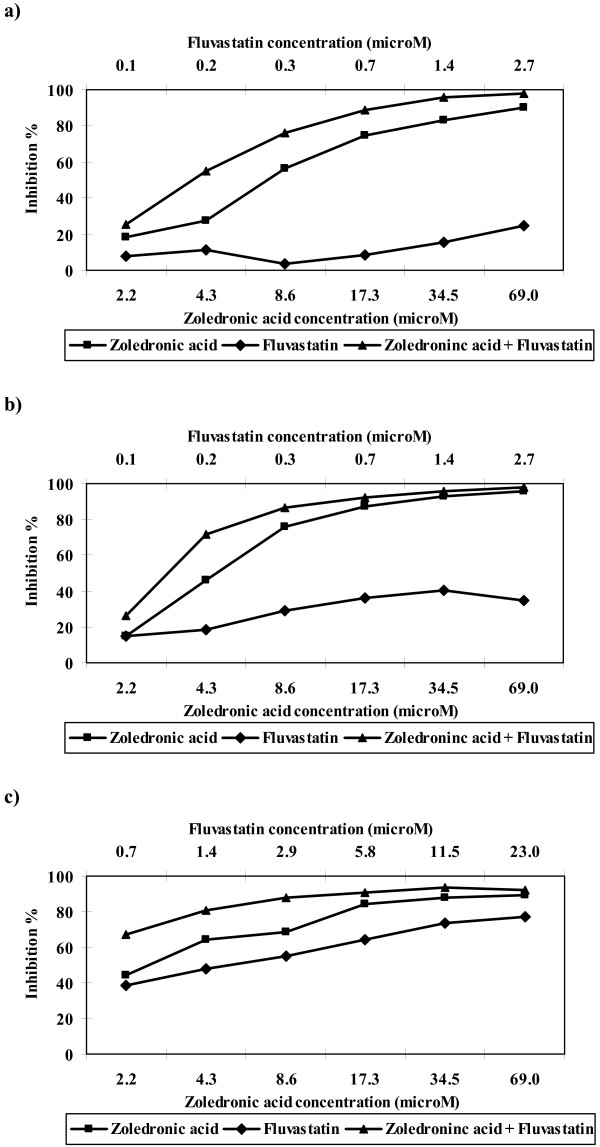
**Median inhibition by zoledronic acid and fluvastatin alone and in combination in a) breast carcinomas tested with fluvastatin concentration A (n = 9); b) ovarian carcinomas tested with fluvastatin concentration A (n = 8) and c) ovarian carcinomas tested with fluvastatin concentration B (n = 14)**. The combination results are expressed as micromolar concentrations of zoledronic acid.

Both zoledronic acid and fluvastatin showed inhibition in the ATP-TCA at all concentrations tested. Zoledronic acid showed greater activity than fluvastatin with lower median Index_SUM _values in all tumours (table 1). Both agents showed a shallow rise in their activity with increasing concentration (figure 2a–c). The degree of inhibition attained by zoledronic acid for breast and ovarian cancer was similar, but fluvastatin appears to be less active against breast cancer in the ATP-TCA in comparison with ovarian tumour samples.

### Effect of combination

**Figure 3:**
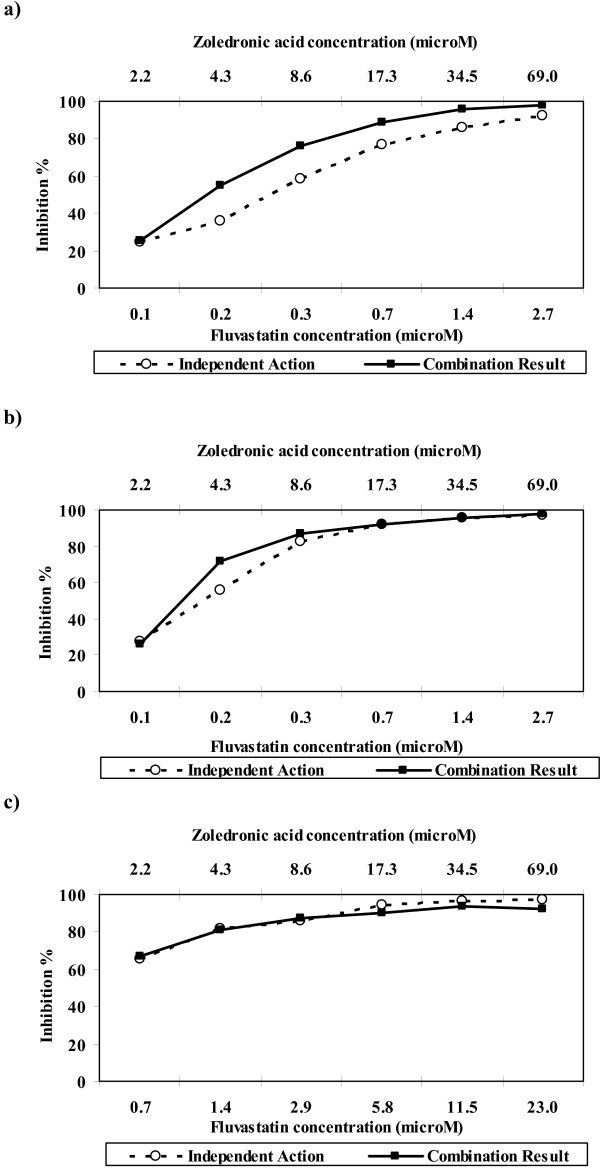
**Median combination analysis showing synergy between zoledronic acid plus fluvastatin in a) breast carcinomas tested with fluvastatin concentration A (n = 9); b) ovarian carcinomas tested with fluvastatin concentration A (n = 8) and c) ovarian carcinomas tested with fluvastatin concentration B (n = 14)**.

The combination of zoledronic acid and fluvastatin showed considerable activity against breast and ovarian cancer. A total of 56% (5/9) breast and 50% (11/22) of ovarian tumours achieved >95% inhibition at clinically achievable concentrations. Figure 3a demonstrates that the combination of zoledronic acid and fluvastatin exhibited synergy in breast samples when the lower concentration of fluvastatin was used (0.1–2.7 μM). In the ovarian samples there was synergy at the lower concentrations (figure 3b) when the lower concentration of fluvastatin was used compared to the higher fluvastatin concentration range where the difference between the independent action and the combination result was negligible (figure 3c). Where there were IC_50 _and IC_90 _values available the CI50 and CI90 values were calculated for each sample using the Chou & Talalay [14] method. For the breast samples the CI50 and CI90 values indicated that, 89% (8/9) and 78% (7/9) showed synergism, 11% (1/9) and 11% (1/9) showed additivity. No samples had a CI50 that indicated antagonism but for the CI90, 1 sample showed antagonism. The median CI50 and CI90 values were 0.6 (synergy) and 0.6 (synergy) respectively. For the ovarian samples treated with the lower fluvastatin concentration, the CI50 and CI90 values indicated that 57% (4/7) and 57% (4/7) showed synergism, no samples had a CI50 that indicated additivity but for the CI90, 1 sample showed additivity, and 43% (3/7) and 14% (1/7) showed antagonism. The median CI50 and CI90 values were 0.8 (synergy) and 0.6 (synergy) respectively. For the ovarian samples treated with the higher fluvastatin concentration, the CI50 and CI90 values indicated that 46% (6/13) and 69% (9/13) showed synergism, 23% (3/13) and 15% (2/13) showed additivity and 31% (4/13) and 15% (2/13) showed antagonism. The median CI50 and CI90 values were 0.9 (additivity) and 0.5 (synergy) respectively.

### Sequential effects

**Figure 4:**
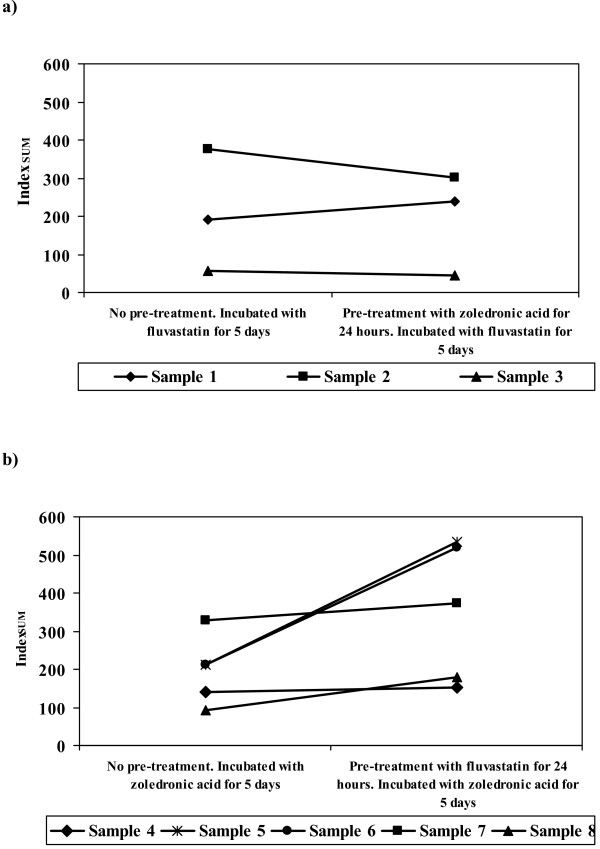
**Schedule dependence of effect of zoledronic acid and fluvastatin compared to singe agent treatment in ovarian tumour derived cells**. Cells were pre-conditioned for 24 hours with a) zoledronic acid or b) fluvastatin and then incubated for 5 days with fluvastatin or zoledronic acid respectively.

The sequential drug experiments show that ovarian tumour cells exposed to fluvastatin exhibit little or no differences in their sensitivity to fluvastatin regardless of whether they have been pre-exposed to zoledronic acid or not (figure 4a). However, in comparison ovarian tumour cells treated with zoledronic acid have a decreased sensitivity to the drug if they have been pre-exposed to fluvastatin (figure 4b). All 5 samples tested with zoledronic acid following pre-exposure to fluvastatin exhibited an increase in their Index_SUM _value compared to cells that had not been pre-exposed to fluvastatin.

### Substrate replacement studies

**Figure 5:**
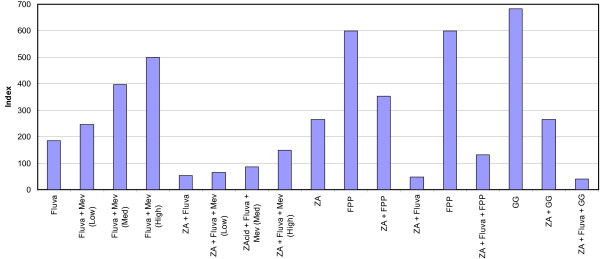
**The effect of mevalonate pathway substrate replacement with mevalonate (Mev) at low, medium and high concentrations (5, 200, and 1537 μM respectively), geranylgeraniol (GG, 10 μM) and farnesyl diphosphate (FPP, 23 μM) on zoledronic acid (ZA) and fluvastatin (Fluva) activity in a recurrent ovarian cancer**.

Experiments were conducted with a single primary ovarian cancer from which large numbers of cells were obtained, allowing a many different experiments to be performed. The effect of mevalonate (5, 200, 1537 μM), geranylgeraniol (10 μM), farnesol (10 μM) and farnesyl diphosphate (FPP, 23 μM) alone on these cells showed no evidence of toxicity. Replacement and pre-incubation experiments (3 and 24 hours) were performed with zoledronic acid and fluvastatin (figure 5). Whereas fluvastatin activity showed reversal by increasing mevalonate concentration, this had little effect on downstream inhibition by zoledronic acid alone or the combination of zoledronic acid + fluvastatin. FPP addition reversed only weakly the effects of fluvastatin (figure 5), and showed minor reversal of the activity of zoledronic acid and zoledronic acid + fluvastatin. Addition of geranylgeraniol and farnesol showed no effects (figure 5). Subsequent experiments were conducted with FPP and mevalonate in two further ovarian cancers, with no effect (data not shown).

## Discussion

The ATP-TCA data show convincing inhibition by both single agents, similar in the case of zoledronic acid to that seen previously [8]. The degree of inhibition by fluvastatin is lower in breast cancer than ovarian cancer cells. Combination of the two drugs is even more effective than zoledronic acid alone and analysis using the Poch *et al*., [13] method indicates that fluvastatin may be more effective when used at a lower concentration. Calculation of the CI50 and CI90 using the Chou & Talalay [14] method demonstrated that there was synergism when zoledronic acid was combined with fluvastatin. Similar data have recently been reported in cell lines by Budman & Calabro [16], showing that the combination of zoledronic acid and fluvastatin is more cytotoxic than either drug alone, and also in myeloma cell lines [17]. However, cell lines can be misleading and it is prudent to examine effects in tumour-derived cells whenever possible [18].

The underlying mechanism for the activity of zoledronic acid and fluvastatin against cancer cells has been suggested to involve Ras prenylation, which is affected by both agents. The combination would be expected to produce an enhanced effect. Preliminary experiments performed by our collaborators have shown inhibition of prenylation of Rap1a in MCF7 cells treated with the single agents (M Rogers, personal communication). Is this is correct, then given before the bisphosphonate, statins would be expected to block entry of mevalonate into the pathway reducing the substrate concentration for the step blocked by zoledronic acid and hence increasing the efficacy of the combination. The sequential data are therefore interesting as they appear to be at variance with this model, and suggest instead that statins are more effective when given after zoledronic acid. This could result from an alternative mechanism.

It has recently been suggested that the generation of an unusual metabolite of ATP, known as ApppI from IPP (figure 1), may be responsible for some of the toxic effects of the N-bisphosphonates [19,20]. If this is the case, greater activity would be expected if the zoledronic acid was given first, followed by the statin to prevent the entry of further mevalonate into the pathway diluting the effect of the ApppI and allowing new pools of FPP to be produced. This explanation is supported by our replacement substrate experiments. In these, mevalonate was able to reverse fluvastatin effects almost completely (at high mevalonate concentrations), while it had no effect on the downstream inhibitor, zoledronic acid. FPP is downstream of the block created by zoledronic acid, but was unable to reverse the zoledronic acid effect, consistent with greater importance of the alternate ApppI mechanism.

Both zoledronic acid and fluvastatin are well tolerated. Indeed, it is likely that a number of patients have been treated with both agents by accident, since the former is indicated for breast cancer patients with metastases and statins are commonly prescribed as cholesterol lowering agents in patients with concomitant ischaemic heart disease. We have been unable to find any evidence of a toxic interaction between N-bisphosphonates and statins in the literature. Zoledronic acid is rapidly excreted by the kidney and has a short half-life such that after a single dose, none is detectable in plasma after 24 hours [21,22]. In human and animal studies, concentrations in bone are around 10 times those in bone marrow, which is in turn in 10 times those in peripheral tissues, where concentrations within the range tested here have been noted after single doses in animals (Novartis – data on file). No human data are available for ascitic fluid or breast tumour tissue, but it is likely that the concentrations used here are active, particularly as the length of exposure will be higher in patients than the assay, which exposes cells to drugs for just 6 days.

The direct effects of zoledronic acid against tumours have also been attributed to immunological effects on γδT-lymphocytes [23] and to effects on angiogenesis [24]. Both effects are of course unlikely within the ATP-TCA as lymphocytes are killed by the selective medium and angiogenesis is not required by cells in culture. Nevertheless, these remain intriguing effects of these drugs and may augment the direct effects on cancer cells seen here and in our previous paper [8]. The combination of these effects may be clinically useful [25], and there is increasing evidence of an effect of zoledronic acid against tumour cells including a recently reported randomised controlled trial in breast cancer [26].

## Conclusion

This study shows that the combination of zoledronic acid and fluvastatin exhibits synergy in breast and ovarian tumour derived cells and that it may have activity against breast and ovarian cancer. A phase II clinical trial is now in preparation.

## Competing interests

IAC is a Director of CanTech Ltd, a University spin-off company which partly funded this study. CMK and IAC have received grant funding from Novartis.

## Authors' contributions

LAK, SG, AF, RR, and SD carried out the experiments described the study, which was designed and analysed by IAC, UR and CMK. All authors read and approved the final manuscript.

## Pre-publication history

The pre-publication history for this paper can be accessed here:

http://www.biomedcentral.com/1471-2407/9/38/prepub
